# Sexual Therapy for Women with Multiple Sclerosis and Its Impact on Quality of Life

**Published:** 2017-01

**Authors:** Maryam Zamani, Azadeh Tavoli, Behjat Yazd Khasti, Neda Sedighimornani, Masood Zafar

**Affiliations:** 1 Counseling Center, Tehran University, Tehran, Iran.; 2 Department of Psychology, Faculty of Educational Sciences and Psychology, Alzahra University, Tehran, Iran.; 3 Department of Sociology, Faculty of Humanity Studies, Isfahan University, Isfahan, Iran.; 4 Department of Psychology, Faculty of Humanities and Social Sciences, University of Bath, Bath, UK.; 5 Counseling Center, Shahed University, Tehran, Iran.

**Keywords:** *Multiple Sclerosis*, *Quality of Life*, *Sexual Therapy*

## Abstract

**Objective:** Multiple Sclerosis (MS) is a disease with a detrimental effect on functional status. The present study investigated the effect of a sexual therapy program on the quality of life (QOL) of women with multiple sclerosis.

**Method:** Women with multiple sclerosis and sexual dysfunction (n = 30) were selected, and were randomly assigned into the treatment (n = 15), or the control groups (n = 15). Participants of the treatment group (n = 15) received 12 weekly sessions of sexual therapy. Participants in both groups completed the Female Sexual Function Inventory (FSFI) and the MS Quality of Life- 54 (MSQOL-54) in the onset of the program and at the end of the program.

**Results:** ANCOVA(s) using pre-test scores as covariate(s) revealed that in comparison to the control condition, MS patients within the treatment group showed a significant improvement in their sexual desire (0.0001), arousal (0.022), lubrication (0.001), orgasm (0.001), satisfaction (0.0001), overall quality of life (0.001), energy (0.023), cognitive function (0.005), and social function (0.001) at the end of the program. In addition, they were less limited in their roles due to the emotional and health problems.

**Conclusion:** The present study revealed that addressing sexual dysfunction in MS patients could improve their quality of life. In the future, this research can extend its results, and apply the same method to men with MS to find whether sexual therapy enhances their quality of life.

Multiple sclerosis (MS) is an immune-mediated disease of central nervous system, causing nerve demyelination and progressive neurodegeneration (1, 2). The cause of this progressive and relapsing disease is unknown, and so is the cure. MS is one of the most common causes of disability in adults (3), and has a significant negative effects on physical and psychological well-being (4, 5). MS is three times more common in women than men (6). In Iran, the prevalence rate of MS is reported to be at least 45 per 100,000 population. According to the recent report, approximately 70% of the MS patients in Iran are female and aged 20 to 40 years. In Iran, Isfahan province has the highest rate of MS (7).

MS has a negative impact on the patient’s quality of life (8). 

Individuals with MS have a lower score on the Health-Related Quality of Life (HRQOL) scale compared to the general population and to other individuals living with a chronic illness (9, 10). 

A number of factors have been identified to influence the quality of life in patients with MS. These include fatigue, psychological symptoms, physical disability (11), rapid MS progression (12), pain (13), and cognitive impairments (14). In addition to these factors, it has been found that the frequent occurrence of sexual dysfunction (SD) in MS patients prominently affects all aspects of their quality of life (15, 16 and 17). 

The relationship between sexual dysfunction and MS is complicated (18, 19). A complex interaction between biological, psychological, and social factors seems to exist, which can be strongly influenced by one’s own sense of self and social competence (20). The occurrence of sexual dysfunction is higher in women with MS (21, 22), ranging from 40- 80% (18, 19). Previous studies have demonstrated that anorgasmia or hyporgasmia, decreased vaginal lubrication, lack of genital sensations, and reduced libido in women with MS were the most common symptoms of SD (18, 23).

For example, Demirkiran et al. (24) found that most patients with MS (nearly 80%) in Turkey complained about primary sexual dysfunctions, and their main issue was reduced libido, which can negatively affect quality of life (25, 26).

In Iran, Merghati- Khoei et al. (27) found that the prevalence of primary sexual problems among Iranian women with MS was 87.1%. Delayed orgasm, spasticity, and worries about the partner’s sexual satisfaction were the most common problems. Similarly, Ashtari et al. (28) demonstrated that the most common sexual dysfunctions among Iranian women were orgasmic problems, and low level of sexual desire. According to these authors (28), MS has a detrimental effect on female sexuality and can severely influence the quality of life (29). Specifically, the onset of multiple sclerosis mainly occurs when individuals are in the prime of their sexual lives (7). Mohammadi et al. (30) stated that factors such as duration and severity of the illness as well as depression significantly contribute to SD in MS female patients. 

In romantic and intimate relationships, sexual satisfaction, or dysfunction is inextricably linked to an individual’s subjective appraisal of his or her quality of life (31); thus, it should be considered whenever we assess the effect of chronic disease or treatments for such diseases (29). In fact, given the importance of quality of life, different methods and approaches have been devised for enhancing it. For example, previous studies indicate that health-promoting behaviours such as physical activity and stress management, can promote the functional status and quality of life of people with MS (32, 33, 34). Furthermore, the effectiveness of relaxation (35), cognitive-behavioural techniques (36), and social support (37) has been demonstrated. 

We believe that since sexual satisfaction significantly influences a dimension of quality of life, sex therapy and education can enhance the quality of life in women with MS. Although sexual function is highly impaired in women with MS (38, 39), it has not received noticeable attention in the literature. More recently, Najafidoulatabad and colleagues (40) examined the effect of yoga exercises on physical and sexual abilities of the Iranian women with MS. Their results indicated that yoga techniques significantly increased the level of sexual satisfaction and physical activities among the women. However, these authors did not assess the quality of life. 

The current study aimed at investigating the effectiveness of sexual therapy on the quality of life in women with multiple sclerosis. The literature seems to suggest that intervention models that focus on information, education, and expression of sexual needs and difficulties are efficacious (18, 31 and 38). For example, Christopherson et al. (25) showed that obtaining written materials about sexual dysfunction encouraged MS patients to discuss their sexual concerns with their spouses, which subsequently enhanced their coping strategies. Foley and colleagues (23) found that education and practice could significantly improve sexual dysfunction. In their study, in the education phase, couples understood their sexual issues and problems. In the rehearsal phase, the therapist educated them about crucial skills for handling sexual difficulties. In the application phase, participants learned how to communicate about their problems without judgment. Similarly, Goldman and Carroll (31) found that elderly couples who were assigned to an educational program revealed a significant sexual satisfaction compared to the control group. 

As women become more informed, they are more able to adjust sexuality, which, in turn, might influence their quality of life. Providing sexual education and assisting women to verbalize their sexual needs in countries such as Iran where women are not very knowledgeable about sexual functioning, and discussing sex is not very common might be a valuable method to enhance sexual satisfaction and the subsequent quality of life.

## Materials and Methods

***Design, Data Collection, and Procedure***

This quasi–experimental study was carried out to examine the effect of sex therapy on the QOL of women with MS. Eligible participants were identified based on the followings: The diagnostic tests for MS; an examination by a neurologist; and an interview conducted by a clinical psychologist who was expert in sexual problems and had previously worked with individuals with sexual dysfunction. Eligible MS patients were invited to participate in the study. Prior to the enrollment, participants were thoroughly informed about the study and its procedure. In addition, written informed consent was obtained from the participants. 

In total, 30 women were diagnosed with MS and sexual dysfunctions (i.e., sexual dysfunctions were determined during the interview and examination). They were randomly assigned into either the treatment or control group. Participants in the treatment group received 12 weekly sessions of sex therapy, while those who were in the control group did not receive any treatment. A clinical psychologist with experience of working with sexual dysfunction interviewed the patients in person and administered the questionnaire. Demographic information including age, educational status, duration of the marriage, and time since diagnosis were collected.

***Participants***

Individuals aged 17 - 45 years who were seeking treatment for MS (attending the Alzahra Hospital in Isfahan) and willing to participate in the trial were recruited. Exclusion criteria were as follows: Patients who had cognitive problems, those who were too ill to participate in the interviews, those who had sexual problems due to marital dissatisfaction and not their MS, and those with prior diagnoses of bipolar disorder, psychotic disorders or drug dependence. Participants were interviewed by a clinical psychologist, and those who did not meet the criteria were excluded from the study. Participants in both the control and intervention groups completed the FSFI and QOL measures at baseline and at the end of the treatment (3 months after the onset of the treatment). The control group did not receive any treatment other than receiving the questionnaires. The sex enhancement program consisted of one training session per week for 12 weeks in a group format. The program was based on a psychosexual therapy (23, 25, 31, 41 and 42). Table 1 Summarizes key elements of the program.

***Research Instruments***

1.     Quality of Life

Quality of life was assessed using the MS Quality of Life- 54 (MSQOL-54), which is a widely used measuring tool. It includes 18 MS-specific questions, as well as, 36 items from the Short-Form Health Survey (43). Vickrey et al. (44) added the aforementioned 18 items to the original SF-36 to address health distress (4 items), sexual function (4 items), satisfaction with sexual function (1 item), overall quality of life (2 items), cognitive function (4 items), energy (1 item), pain (1 item), and social function (1 item). The MSQOL-54 instrument comprises 52 items distributed into 12 multi-item scales, and two single items. Previous findings indicate that the Iranian version of the MSQOL-54 is a reliable and valid measure of the quality of life in patients with MS (45).

2.      Female Sexual Function Inventory

Sexual dysfunctions were measured by the Female Sexual Function Index (FSFI). This is a known instrument that assesses sexual function in women in six domains: Desire, arousal, lubrication, orgasm, satisfaction, and pain during sexual intercourse (23). The FSFI scores below 24 indicate sexual dysfunction. The Iranian version of this measure has demonstrated a good validity and reliability (46). 

***Statistical Analysis***

Descriptive statistics were used to examine the sample characteristics. Pre-test scores were assessed using analyses of covariance (ANCOVA). QOL scores at the pretest with other pretest measures were used as covariates to control for the pretest effect and initial differences between the two groups. A p-value < 0.05 was regarded to denote statistical significance.

## Results

***Patients' Characteristics***

A total of 30 MS patients with sexual dysfunction (treatment group n = 15, control group n = 15) participated in the study. Both groups were similar in terms of demographics and clinical characteristics. The detailed demographic and clinical data are summarized in Table 2.

**Table1:** Outline of the Sexual Therapy Sessions

**Sessions 1-2**	Basic ground rules and educational information about sexual anatomy, the physiology of sexual response, moisturizers, vaginal dilators, enhancing arousal and increasing low desire, and sexual myths or misconceptions related to sexual responses were reviewed. Assigned exercises included relaxation, Kegel exercises, and self-exploration. Homework was assigned.
**Sessions 3-8**	The previous’ week's homework was reviewed. Instructions about the initiation of sexual activities or refusal of them were given. There were lessons about expressing sexual tastes and preferences. During this time, intercourse was banned and the emphasis was on non-genital, then on genital caressing. Homework was assigned.
**Sessions 9-11**	The previous’ week's homework was reviewed. Specific skills about the self and interpersonal pleasuring to facilitate sexual enjoyment and expression, such as: body awareness, physical sensation scan, and progressive muscle relaxation were taught. Participants learned about receiving prolonged sexual stimulation without feeling obligated to reciprocate immediately. Intercourse was resumed during this period. Homework was assigned.
**Session 12**	Homework from the previous week was reviewed. Reevaluation of the participants’ progress, evaluation of the gains produced by the program, and individuals’ problems were mentioned. Effective measures to overcome these issues were discussed.

**Table2:** Demographic and Clinical Characteristics of Patients in the Study

**Variables**	**Treatment Group (N = 15)**	**Control Group (N = 15)**
	**M (SD)**	**M (SD)**
Age (years)	37(5.87)	34(8.08)
Duration of marriage (years)	17.2(6.96)	13.9(9.73)
Time since diagnosis (years)	5.2(3.05)	3.86(4.5)

Note: SD = Standard Deviation

**Table3:** Scores for the MSQOL-54 and FSFI Scales in Patients with MS in the Treatment and Control Groups

**Measures**	**Treatment Group**			**Control Group**	
	**Pre-intervention** **Mean (SD)**		**Post- intervention** **Mean (SD)**	**Pre-test** **Mean (SD)**	**Post-test** **Mean (SD)**
**MSQOL54**					
Emotional well-being		59.2 (12.7)	63.4(7.8)	59.4(10.6)	58.13(9.3)
Physical health		66.69 (22.8)	66.33(19.5)	66.66(20.6)	58.3(19.3)
Role limitations due to physical problems		36.6(38.8)	30(42.4)	38.3(36.4)	21.6(32.5)
Limitations due to emotional problems		57.7 (44.4)	62.2 (45.1)	55.5(41.1)	35.5(42.6)
Health distress		63.3 (14.8)	67 (13.06)	62.6(11.3)	61(11.6)
Health perceptions		50.3 (16.1)	53.3 (14)	51 (13.2)	52 (12.2)
Mental health		58.9 (18)	64.3 (15.5)	59.4 (14.5)	53.6 (14.6)
Social functions		66.1 (17)	74.4 (13.1)	70 (13.6)	66.6 (11.3)
Pain		58.4 (17.3)	74 (12.08)	58 (20.1)	54.4 (18.45)
Energy		46.8 (15.4)	53.6 (15.5)	50.9 (15.3)	50.9 (13.8)
Cognitive function		53.6 (14.4)	58 (12)	62.3 (14.2)	59.2 (16)
Overall quality of life		6.9 (13.5)	71.7 (10)	59.8 (10.6)	60.3 (9.3)
Satisfaction with sexual function		33.3 (24.3)	71.7 (12.9)	28.3 (26.5)	23.3 (17.5)
Sexual function		51.1 (19.1)	79.4 (13.6)	49.4 (16.8)	45.5 (13.3)
**FSFI**					
Desire		2.48 (1/1)	3.88 (0.6)	2.7 (1.2)	2.76 (1.1)
Arousal		3.02 (1)	3.8 (0.6)	3.6 (0.9)	2.7 (0.01)
Lubrication		3.8 (1.07)	4.5 (0.8)	4.1 (1.7)	4.1 (1.5)
Orgasm		3.3 (1.03)	4.4 (0.6)	3.1 (1.3)	3.5 (0.9)
Satisfaction		3.6 (1.1)	4.5 (0.4)	3.6 (0.6)	3.5 (0.9)
Pain		4.7 (1.2)	5.4 (0.6)	4.8 (1.4)	4.5 (1.8)
Overall FSFI		3.45 (1.08)	4.41 (.6)	3.65 (1.18)	3.51 (1.03)

Note: MSQOL54 = MS Quality of Life-54; FSFI = Female Sexual Function Inventory

***Sexual Function and Quality of Life***

Table 3 depicts the scores on the sexual function and quality of life scales for the treatment and control groups. Comparison of the sexual function scores between MS patients in the treatment and control groups, using analysis of covariance (ANCOVA) at the baseline and after intervention, indicated that patients within the treatment group showed improvements in the different aspects of sexual function such as desire (0.0001), arousal (0.022), lubrication (0.001), orgasm (0.001), satisfaction (0.0001), and pain (0.026). 

In addition, the analysis showed that there were some improvements within the treatment group in most aspects of the quality of life as measured by the MSQOL-54 such as satisfaction with sexual function (< 0.001), overall quality of life (<0.001), energy (0.023), improved cognitive function (0.005), improved sexual function (< 0.001), and improved social function (0.001) compared to the control group. Furthermore, individuals in the treatment group were less limited in their roles because of the emotional problems (0.014), health distress (0.023), and pain (< 0.001) than the control group. However, the differences between role limitations due to physical problems (0.315) and health perceptions 

(0.22) were not significant between the two groups (Table 4).

## Discussion

The purpose of this study was to examine the effect of sexual therapy and education on the quality of life of women with MS. The results from this study revealed that participation in the sexual therapy program could positively influence the quality of life of women with MS. 

Prior intervention studies support the positive effects of exercise and physical activity (47), progressive muscle relaxation techniques (35), and social support (37) on the quality of life. This study adds to the literature by addressing the association between sexual function and quality of life in patients with MS. McCabe et al. (48) stated that since the onset of multiple sclerosis, about one-third of their respondents reported a relationship breakdown, while about one-third indicated that their relationship has become stronger. 

**Table4:** The Results of ANCOVAs for the MSQOL-54 and FSFI Scales Using the Baseline Scores as Covariate

	**F-value**	P*
**MSQOL-54**		
Emotional well-being	7.4	0.011
Physical health	3.4	0.076
Role limitations due to physical problems	1.05	0.315
Limitations due to emotional problems	6.9	0.014
Health distress	5.7	0.023
Health perceptions	1.5	0.22
Mental health	15.8	< 0.001
Social functions	12.5	0.011
Pain	30	< 0.001
Energy	5.8	0.023
Cognitive function	9.4	0.005
Overall quality of life	25.2	< 0.001
Satisfaction with sexual function	14.1	< 0.001
Sexual function	84.2	< 0.001
**FSFI**		
Desire	18.1	< 0.001
Arousal	1.5	0.022
Lubrication	14.9	0.011
Orgasm	16.5	< 0.001
Satisfaction	5.5	0.026

MSQOL: Multiple Sclerosis Quality of Life-54 Questionnaire

Expressions of concern, love, and understanding are crucial in the eyes of MS patients, whereas attempts to minimize or maximize the effects of multiple sclerosis, and its associated symptoms are seen as the most unhelpful responses (49). We understand that multiple sclerosis results in increasing dependence upon others for both social and practical support. Since the family is often the closest and the obvious source of support, the development of multiple sclerosis has a significant effect on family dynamics (50). 

Thus, in the future research, it might be beneficial to include partners in the treatment process; especially, since the expression of sexual function usually relies on the presence and cooperation of a partner (48). Education is beneficial for boosting sexual function and quality of life; therefore, increasing awareness about sex and sexuality might enhance couples’ relationship, interaction, and life satisfaction.

Sexual dysfunction in multiple sclerosis is described as having three levels of influencing factors (51). First, primary sexual dysfunction, which happens because of multiple-sclerosis-related neurological changes, directly affects sexual feelings or sexual response. The primary symptoms include decreased libido, altered genital sensation, decreased vaginal lubrication, and decreased frequency or intensity of orgasm. Secondary sexual dysfunction includes multiple sclerosis-related physical changes and symptoms, which affect the sexual response indirectly. The secondary symptoms include fatigue, muscle tightness, weakness, spasms, bladder and bowel dysfunction, bad coordination, difficulties with mobility, side effects of multiple sclerosis medications, cognitive difficulties, numbness, pain, burning, or discomfort in non-genital areas of the body. The so-called tertiary sexual dysfunction is caused by psychological, emotional, social, and cultural aspects of multiple sclerosis that impact sexuality. Tertiary symptoms include negative changes in self-image, mood, or body image, depression and anger, feeling less sexy or attractive, fear of being rejected sexually, difficulties in communicating with one’s partner, fear of isolation and abandonment, guilt, changing gender roles, and feelings of dependency (30, 51). In the current study, we focused on psychological issues to treat sexual dysfunction in women with MS. The results revealed that patients in the treatment group reported enhancements in their desire, arousal, lubrication, orgasm, satisfaction, and decrease in pain during sex.

Furthermore, the analysis revealed that quality of life, as measured by the MSQOL-54, in the treatment group improved significantly compared to the control group. In other words, the sex therapy program was effective in improving the level of quality of life in women with MS. ANCOVAs, using pre-test scores as covariates, revealed that the treatment group reported more satisfaction with sexual function, better quality of life, more energy and better cognitive function, improved sexual, and social functions compared to the control group. 

However, on the QOL dimensions that were assessed by the MSQOL54, two variables of health perception and role limitations were not significantly affected by sex therapy. It seems that although sex therapy has an impact on the quality of life and psychological aspects of the MS patients, it might not significantly influence their health perception and physical limitations because of the physical disabilities and dysfunctions that they experience.

The goals for individuals with MS are to enhance their quality of life (in all aspects) and slow the progression of the disease. As with any chronic physical and psychological problem, managing multiple sclerosis requires various combinations of therapies and treatment models (52). In clinical practice, due to the obvious sensitivity of the issue, patients might be reluctant to talk about their sexual problems, and many physicians may feel uncomfortable or inadequately trained to discuss sexual issues with their patients. However, the vast majority of the patients believe that it is appropriate for physicians to address sexual problems within the context of routine health assessment (53). Nonetheless, a recent survey (54) indicates that while sexual dysfunctions among MS patients are common, only one-third of these patients have discussed their sexual concerns with their healthcare providers in the past year. Our study demonstrated the importance of sexual satisfaction in patients with MS and its effect on the quality of life. Educating MS patients about sexual function and dysfunction is highly important and needs further investigation.

## Limitations

Several limitations of the study should be noted. First, as mentioned earlier, we excluded patients with prior diagnoses of bipolar disorder, psychotic disorders, or drug dependence. Nevertheless, since we did not control for the possible presence of disorders such as depression, panic disorder, and generalized anxiety disorder, these comorbidities might have influenced the results. The use of medications such as antidepressant and anticholinergic was likely to contribute to sexual dysfunction in patients with MS. Furthermore, the sample size in this study was small, so the results should be regarded with cautious. In addition, this research was limited to women with mild to moderate disability, and this may preclude generalization of the results to those patients with more severe illness or male patients. Therefore, additional research is needed to explore the influence of sex therapy on the quality of life of people with more aggressive form of MS. Despite the mentioned limitations, the results of this study represent encouraging evidence that sex therapy could enhance the quality of life of MS patients. We suggest that future studies investigate this issue further and find whether sex therapy could have a positive long-term effect.

## Conclusion

In conclusion, the findings from the present study demonstrated that patients who were in the treatment group and received sex therapy evaluated their quality of life better in comparison to those who were in the control group. This suggests that sex education can enhance the quality of life and the well-being of people with MS. This study emphasizes that healthcare providers should consider sex-enhancing therapies for patients with MS as a strategy to improve their quality of life.
